# Bayesian spatial modelling of intimate partner violence and associated factors among adult women and men: evidence from 2019/2020 Rwanda Demographic and Health Survey

**DOI:** 10.1186/s12889-023-16988-8

**Published:** 2023-10-20

**Authors:** Innocent Maposa, Halima S. Twabi, Zvifadzo Matsena-Zingoni, Jesca M Batidzirai, Geoffrey Singini, Mohanad Mohammed, Alphonce Bere, Kabelo Kgarosi, Nobuhle Mchunu, Portia Nevhungoni, Maureen Moyo-Chilufya, Oludoyinmola Ojifinni, Alfred Musekiwa

**Affiliations:** 1 Division of Epidemiology & Biostatistics, School of Public Health, Faculty of Health Sciences, University of the Witwatersrand, Johannesburg, South Africa; 2 Department of Mathematical Sciences, School of Natural and Applied Sciences, University of Malawi, Zomba, Malawi; 3 School of Mathematics, Statistics, and Computer Science, University of KwaZulu-Natal, Pietermaritzburg, South Africa; 4 Department of Mathematical and Computational Sciences, University of Venda, Thohoyandou, South Africa; 5 School of Health Systems and Public Health, Faculty of Health Sciences, University of Pretoria, Pretoria, South Africa; 6 Biostatistics Research Unit, South African Medical Research Council, Durban, South Africa; 7 Centre for the AIDS Programme of Research in South Africa (CAPRISA), Statistics, Durban, South Africa; 8 Biostatistics Research Unit, South African Medical Research Council, Pretoria, South Africa; 9 School of Clinical Medicine, Faculty of Health Sciences, University of the Witwatersrand, Johannesburg, South Africa; 10 Center for Biomedical Modelling, Department of Psychiatry and Biobehavioural Sciences, David Geffen School of Medicine, University of California, Los Angeles, CA USA; 11 Division of Epidemiology & Biostatistics, Department of Global Health, Faculty of Medicine and Health Sciences, Stellenbosch University, Cape Town, South Africa

**Keywords:** Intimate partner Violence, Bayesian, Spatial effects, Choropleth, Mapping cluster, Fixed effects, Generalized additive mixed effects model

## Abstract

**Background:**

Intimate partner violence (IPV) remains a global public health concern for both men and women. Spatial mapping and clustering analysis can reveal subtle patterns in IPV occurrences but are yet to be explored in Rwanda, especially at a lower small-area scale. This study seeks to examine the spatial distribution, patterns, and associated factors of IPV among men and women in Rwanda.

**Methods:**

This was a secondary data analysis of the 2019/2020 Rwanda Demographic and Health Survey (RDHS) individual-level data set for 1947 women aged 15–49 years and 1371 men aged 15–59 years. A spatially structured additive logistic regression model was used to assess risk factors for IPV while adjusting for spatial effects. The district-level spatial model was adjusted for fixed covariate effects and was implemented using a fully Bayesian inference within the generalized additive mixed effects framework.

**Results:**

IPV prevalence amongst women was 45.9% (95% Confidence interval (CI): 43.4–48.5%) while that for men was 18.4% (95% CI: 16.2–20.9%). Using a bivariate choropleth, IPV perpetrated against women was higher in the North-Western districts of Rwanda whereas for men it was shown to be more prevalent in the Southern districts. A few districts presented high IPV for both men and women. The spatial structured additive logistic model revealed higher odds for IPV against women mainly in the North-western districts and the spatial effects were dominated by spatially structured effects contributing 64%. Higher odds of IPV were observed for men in the Southern districts of Rwanda and spatial effects were dominated by district heterogeneity accounting for 62%. There were no statistically significant district clusters for IPV in both men or women. Women with partners who consume alcohol, and with controlling partners were at significantly higher odds of IPV while those in rich households and making financial decisions together with partners were at lower odds of experiencing IPV.

**Conclusion:**

Campaigns against IPV should be strengthened, especially in the North-Western and Southern parts of Rwanda. In addition, the promotion of girl-child education and empowerment of women can potentially reduce IPV against women and girls. Furthermore, couples should be trained on making financial decisions together. In conclusion, the implementation of policies and interventions that discourage alcohol consumption and control behaviour, especially among men, should be rolled out.

**Supplementary Information:**

The online version contains supplementary material available at 10.1186/s12889-023-16988-8.

## Background

Intimate partner violence (IPV) refers to all forms of behaviour within an intimate relationship that causes physical, sexual or psychological harm, including acts of physical aggression, sexual coercion, psychological abuse, and controlling behaviours [1, 2]. It has been recognized globally as an important public health problem and a huge violation of human rights [3] which has been associated with significant morbidity and mortality as well as health and socioeconomic effects among both women and men [4, 5].

Intimate partner violence is an important public health problem and is associated with several reproductive health issues including increased risk of human immunodeficiency virus (HIV) and sexually transmitted infections (STIs), mental health, unintended pregnancy and pregnancy loss [6–8]. Intimate partner violence against women has an estimated global prevalence of 30% [3]. The prevalence of IPV however varies across societies, socioeconomic groups, races and sexes depending on various factors [9, 10]. Previously described risk factors associated with IPV against women include a history of exposure to violence (parental history of spousal violence), marital discord or dissatisfaction, difficulties in communicating between partners and male controlling behaviours between partners, lower levels of education, and a history of exposure to child maltreatment [3, 11]. A prevalence study conducted by the WHO in ten countries with varying income levels uncovered a substantial disparity in the occurrence of IPV. The research revealed that approximately 15%–71% of women had experienced IPV at least once in their lifetime [6]. The same study showed prevalence was especially high in low-income countries. On the other hand, IPV against men is not uncommon but is usually neglected, hence, limited research on IPV against men [12–18].

The prevalence of IPV is particularly high in Sub-Saharan African countries, especially where there are significant disparities between men and women in terms of income, land ownership, and legal rights. Most countries in the Sub-Saharan African region have assimilated IPV practice into their religious and socio-cultural traditions [19–22]. Interventions that could aid in the reduction of IPV among women include community initiatives such as the promotion of sports for women [23] and equitable land-owning policies for both men and women [24]. These would assist in achieving the fifth United Nations Sustainable Development Goal (SDG) which aims to achieve gender equality and empower all women and girls (UN-women) [25, 26]. The second target of this SDG alludes to ending all forms of violence against women and girls by 2030 (UN-women), which includes IPV.

Prevalence and factors associated with IPV against women in Rwanda have been well described [27], however, there is insufficient data on IPV against men. There has been growing interest in spatial analysis techniques in recent years as a tool for an in-depth understanding of public health problems including identifying IPV hotspots, spatial distribution, patterns and effects. Intimate partner violence has been shown in some studies to be spatially distributed [1, 27, 28], however none of the studies have used flexible structured additive regression models to estimate district spatial effects while adjusting for other covariates in studying IPV against men and women. A recent study [13] using the 2019/2020 Rwandan Demographic and Health Survey (RDHS) dataset looked at the factors associated with IPV against women and men but did not consider spatial heterogeneity of IPV. Health and social issues are known to have spatial dependency; ignoring to take into account such random effects may result in biased estimates [29].

This study estimates the prevalence of IPV against women aged 15–49 years and men aged 15–59 years as well as the associated factors by applying Bayesian structured additive logistic regression modelling to the 2019/2020 Rwanda DHS dataset. The spatial heterogeneity of IPV against men and women is determined and the contrasts between the two are highlighted.

## Methods

### Study design and setting

This study was a secondary data analysis of the Rwanda Demographic and Health Survey (RDHS) cross-sectional study. Rwanda is a low-income country with a population of about 13.7 million [30]. It shares borders with Burundi, Uganda, the Democratic Republic of Congo (DRC) and Tanzania. Rwanda has two major ethnic groups, that is, the Hutu and Tutsi who account for more than four-fifths of the population, highlighting a more homogeneous population compared to other African countries. About 75% of this population live in rural areas. The country is divided administratively into 5 regions with the capital in Kigali and 30 districts nested in these regions. The country’s economy relies on agriculture, tourism, mining and donor support [31, 32].

### Source of data and sample

The study population was women 15–49 years and men 15–59 years who lived in Rwanda at the time of the survey. The detailed sampling methods are described [33] elsewhere. Briefly, a stratified, two stage cluster random sampling of households was done. Among all the respondents in the survey, 1947 women and 1371 men consented and responded to the domestic violence survey questions and these were included in our study.

The dataset was obtained from the 2019/2020 Rwandan Demographic and Health Survey. A request to use the dataset was made to the Demographic and Health Survey (DHS) team (ICF International-DHS) and permission was granted. The RDHS was reviewed and approved by the Rwanda National Ethics Committee (RNEC) and the ICF Institutional Review Board. The DHS is a nationally representative cross-sectional survey that among other things monitors domestic violence and other public health indicators. The domestic violence module in the DHS, from which our data is derived, used a shortened and modified conflict tactics scale (CTS) [34] to measure different forms of IPV [7, 35] and domestic violence in general for both women 15–49 years and men 15–59 years.

#### Measurements and covariates

In this study, three forms of spousal violence were considered, that is emotional, physical and sexual violence to generate the outcome. The outcome was a binary variable, which measures IPV in three dimensions, from the questions; (i) Ever experienced physical violence? (ii) Ever experienced sexual violence? and (iii) Ever experienced emotional violence? We also generated other variables to capture; (1) the couple’s financial decision-making strategies i.e. husband making decisions alone or with a partner, and (2) a controlling behaviour. Background characteristic variables such as region, place of residence, age, respondent’s level of education, partner’s educational level, respondent’s age and wealth level were considered as covariates. In addition, age difference, from the respective ages of partners/couples in the dataset was generated from existing variables. Since IPV may be associated with the location of women’s or men’s residences, the spatial heterogeneity was estimated at the district level and these spatial effects were modelled simultaneously with linear and nonlinear effects [1, 27, 36, 37]. The district was selected to provide a low administrative IPV spatial estimate that allows the implementation of target interventions for both men and women to reduce IPV rates. The analysis was performed separately for both women and men.

### Bayesian structured additive logistic regression model

Let ${y}_{ij}$ be the IPV status for a woman or man $i$ in district $j$. ${y}_{ij}=1$ if the woman or man $i$ in district $j$ experienced some form of IPV and ${y}_{ij}=0$ otherwise. A vector ${X}_{ij}=({x}_{ij1},{x}_{ij2},\dots ,{x}_{ijp}{)}^{{\prime }}$ contains $p$ continuous covariate random variables and ${Z}_{ij}=({z}_{ij1},{z}_{ij2},\dots ,{z}_{ijr}{)}^{{\prime }}$ contains some r categorical variables. In our study, $p=1$ and $r=5$. This study assumed that the dependent variable, ${y}_{ij}$ is a Bernoulli-distributed random variable with ${y}_{ij}|{p}_{ij}\sim Bernoulli({p}_{ij})$ with an unknown $E\left({y}_{ij}\right)={p}_{ij}$, being related to the covariates through the logit link function

$$g\left({p}_{ij}\right)={X}_{ij}^{{\prime }}\beta +{Z}_{ij}^{{\prime }}\theta \hspace{0.25em}\hspace{0.25em}\hspace{0.25em}\hspace{0.25em}\hspace{0.25em}\hspace{0.25em}\hspace{0.25em}\hspace{0.25em}\hspace{0.25em}\hspace{0.25em}\hspace{0.25em}\hspace{0.25em}\hspace{0.25em}\left(1\right)$$

where $\beta$ is the $p$ dimensional vector of coefficients for the continuous random variables, and $\theta$ is an $r$ dimensional vector of coefficients for categorical random variables. To assess both the non-linear effects of continuous random variables and spatial autocorrelation, we employed a semi-parametric model which utilizes a penalized regression approach [38]. The penalized regression approach is a non-parametric method of ordinary least squares (OLS) which relaxes the highly restrictive linear predictor for a versatile semi-parametric predictor [38, 39]. The flexible semi-parametric predictor is defined by:

$$g\left({p}_{ij}\right)=\sum _{v=1}^{p}{f}_{v}\left({x}_{ijv}\right)+{f}_{spat}\left({s}_{j}\right)+{Z}_{ij}^{{\prime }}\theta \hspace{0.25em}\hspace{0.25em}\hspace{0.25em}\hspace{0.25em}\hspace{0.25em}\hspace{0.25em}\hspace{0.25em}\hspace{0.25em}\hspace{0.25em}\hspace{0.25em}\hspace{0.25em}\left(2\right)$$

where ${f}_{v}(.)$ represents the non-linear twice differentiable smooth function for the continuous covariates and ${f}_{spat}\left({s}_{j}\right)$ is the variable that denotes the spatial effects for each region. In our study, as in [38], we consider a convolution approach to the spatial effects. The assumption is that the spatial effects can be decomposed into two pure components, that is, spatially structured and spatially unstructured effects given as ${f}_{spat}\left({s}_{j}\right)={f}_{str}\left({s}_{j}\right)+{f}_{unstr\left({s}_{j}\right)}$. The final model for our study then becomes:

$$g\left({p}_{ij}\right)=\sum _{v=1}^{p}{f}_{v}\left({x}_{ijv}\right)+{f}_{str}\left({s}_{j}\right)+{f}_{unstr}\left({s}_{j}\right)+{Z}_{ij}^{{\prime }}\theta \hspace{0.25em}\hspace{0.25em}\hspace{0.25em}\hspace{0.25em}\hspace{0.25em}\hspace{0.25em}\hspace{0.25em}\hspace{0.25em}\hspace{0.25em}\hspace{0.25em}\left(3\right)$$

### Statistical methods

Statistical analysis was performed in STATA version 17 (StataCorp, College Station, TX, USA) and R version 4.1.0. The data were survey set to adjust analysis results for design and other survey effects. Weighted percentage frequencies were used to estimate the prevalence of IPV and associated 95% confidence intervals (CI). Cross-tabulations and chi-square tests were used for the bivariate analysis of categorical variables. Mean and standard deviation or median and interquartile ranges were used to summarize continuous variables depending on the distribution. Univariate and multivariable logistic regression models (conventional methods) were used to determine factors associated with IPV in both women and men. To fit the Bayesian structured additive logistic regression model, variables to be included in the model were selected using nominal p-value of 0.2 or less in the conventional multivariable logistic regression and some were literature informed. Significant factors in the multivariable model were adjusted for in the spatial model. Spatial distribution and patterns including Global Moran I and local Moran I for assessing autocorrelation and local clustering were assessed using R v 4.1.0. A conditional autoregressive (CAR) generalized structured additive logistic regression model with the binomial link was fitted using the *BayesX* R package accounting for spatial effects. In addition, non-linear effects of experiencing IPV for some continuous covariates were also assessed. All tests were two-sided and a p-value of less or equal to 0.05 was considered to indicate statistical significance. The 95% credible intervals are reported with adjusted odds ratios (aOR) for the full Bayesian inference.

### Ethical considerations

We sought permission to use DHS data from the DHS program via their website and agreed to all standards and laws applicable in accessing and utilizing DHS data. The Rwandan DHS was ethically approved by the Rwandan Health Research Committee, Institutional Review Board of ICF Macro, and Centre for Disease and Control (CDC) in Atlanta, GA, USA, and Prevention IRB [40].

## Results

### Baseline characteristics

A total of 1947 women were questioned on domestic violence. At least half of the women were aged 35–49 years, while most of the women resided in rural areas (83.5%). More than 60% of the women had primary education and most of them were from the eastern region of Rwanda with the least coming from Kigali (13.4%). Utmost 20% were from the richest households and more than 80% of the women were employed in the past 12 months. For marital status, a majority of the women were not living together with their partner and more than 60% of the study participants had partners who completed primary education. Almost 50% of the women stated that their partners had a controlling behaviour and that they did not make financial decisions together with their partners (68.5%). Most of the women had partners who were older than them (79.6%) and partners that consumed alcohol (63.2%). Majority of the participants were not currently pregnant (91.6%), see Table 1.

**Table 1:** Baseline characteristics of women and men who participated in the domestic violence module in the survey, Rwanda 2019/2020

Characteristics	Women (N = 1947) N (%)	Men (N = 1371) N (%)
**Socio-demographic**		
**Age group in years**		
15–24	219 (11.0)	52 (3.7)
25–34	834 (38.3)	464 (29.6)
35–49	894 (50.7)	654 (48.2)
50 above		201 (18.5)
**Residence**		
Urban	369 (16.5)	271 (15.5)
Rural	1578 (83.5)	1100 (84.5)
**Highest education**		
None	263 (14.0)	184 (14.0)
Primary	1277 (63.6)	957 (70.6)
Secondary	327 (17.4)	170 (11.7)
Higher	80 (5.0)	60 (3.7)
**Region**		
Kigali	212 (13.4)	151 (12.6)
South	477 (21.6)	332 (20.8)
West	456 (21.9)	319 (22.8)
North	317 (14.9)	234 (16.8)
East	485 (28.1)	335 (27.1)
**Wealth index**		
Poorest	457 (20.5)	280 (18.2)
Poorer	406 (19.6)	277 (20.3)
Middle	358 (19.1)	322 (24.4)
Richer	392 (21.1)	265 (20.0)
Richest	334 (19.7)	227 (17.1)
**Employed in the past 12 months**		
No	286 (14.2)	12 (1.5)
Yes	1661 (85.8)	1359 (98.5)
Partners’ educational status*		
None	231 (13.8)	-
Primary	1155 (67.8)	-
Secondary	198 (12.2)	-
Higher	82 (6.1)	-
Missing	281 (14.4)	-
**Marital status**		
Not together	1017 (52.9)	898 (67.1)
Living with partner	649 (31.1)	473 (32.2)
**Controlling behaviour**		
No	900 (47.6)	747 (54.9)
Yes	1047 (52.4)	624 (45.1)
**Financial decisions**		
Don’t decide together	1157 (68.5)	206 (16.3)
Decide together	509 (31.5)	1112 (83.7)
Missing	281 (14.4)	53 (3.9)
**Spouse related**		
**Partner age difference**		
Same	146 (7.5)	120 (8.8)
Women older	268 (12.9)	266 (19.4)
Man older	1533(79.6)	985 (71.9)
**Partner drinks alcohol**		
No	717 (36.8)	929 (67.8)
Yes	1230 (63.2)	442 (32.2)
**Currently/Wife pregnant**		
No or unsure	1770 (91.6)	1178 (89.8)
Yes	177 (8.4)	139 (10.2)
*Number of partners		
None		53 (4.0)
one		1273 (92.3)
Two or more		45 (3.7)

*Variables missing in either the men’s or women’s datasets

A total of 1371 men were interviewed about domestic violence with a majority aged between 35 and 49 years. Most of the men (80%) resided in rural areas and had completed their primary education. Almost 25% of the men were from the eastern region with the least from the Kigali region. Less than 20% were from the richest households, while a majority were employed in the past 12 months. More than 60% of the men were not living with a partner and the majority made financial decisions together with their partners. Most of the men were older than their partners (71.9%). Utmost 70% of the study participants had partners who consumed alcohol. The majority of the men had partners who were not currently pregnant (89.8%) and almost all the men had one partner (92.3%), see Table 1.

Out of the 1947 women interviewed on domestic violence, 668 (34.6%) reported experience of emotional violence, 711 (36.3%) physical violence and 297 (15.6%) sexual violence. Overall, 895 (45.9%) experienced all forms of violence. For the men, 216 (16.7%) experienced emotional violence, 114 (8.7%) physical violence and 14 (1.1%) sexual violence. In total, 245 (18.4%) of the men experienced all forms of violence, see Table 2.

**Table 2:** All forms of violence with subitems [women & men]

Forms of partner violence experienced		Women 15–49 years (N = 1947)		Men 15–59 years (N = 1371)	
		N (%)	95% CI	N (%)	95% CI
Emotional violence:	Yes	668 (34.6)	32.2–37.0	216 (16.7)	14.5–19.1
	No	1279 (65.4)	63.0-67.8	1155 (83.3)	80.9–85.5
Physical violence:	Yes	711 (36.3)	33.9–38.7	114 (8.7)	7.1–10.6
	No	1236 (63.7)	61.3–66.1	1257 (91.3)	89.4–92.9
Sexual violence:	Yes	297 (15.6)	13.7–17.8	14 (1.1)	0.6-2.0
	No	1650 (84.4)	82.2–86.3	1357 (98.9)	98.0-99.4
Any type of intimate partner violence:	Yes	895 (45.9)	43.4–48.5	245 (18.4)	16.2–20.9
	No	1052 (54.1)	51.5–56.6	1126 (81.6)	79.1–83.8

### Spatial patterns and distribution of IPV

**Fig. 1:**
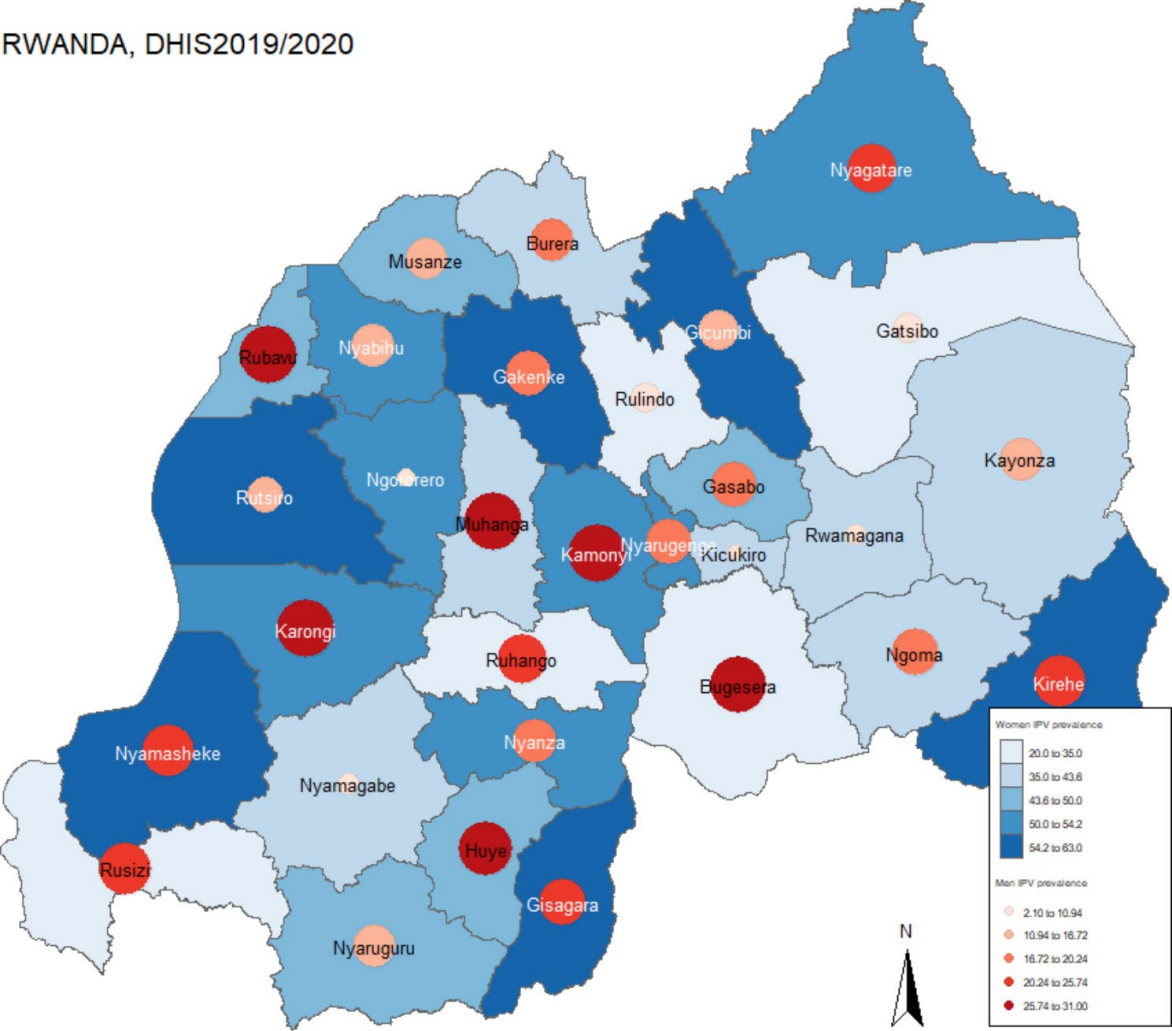
Women and Men IPV prevalence in Rwanda

Figure 1 shows the spatial distribution of IPV prevalence for both men and women in Rwanda. High IPV prevalence among women is noted in Gakenge and Gicumbi districts in the Northern province; Gisagara, Kamonyi, Nyanza and Nyaruguru districts in the Southern province; Kirehe and Nyagatare districts in the Eastern province and Karongi, Ngororero, Nyabihu, Nyamasheke and Rutsiro districts in the western province. Kigali districts had lower prevalence of IPV perpetrated to either women or men. For men, high IPV was noted in Gisagara, Huye, Kamonyi, and Muhanga districts in the Southern province; Bugesera, Kirehe, Ngoma and Nyagatare in the Eastern province; and Karongi, Nyamasheke, Rubavu and Rusizi in the Western province. Of the 30 districts in Rwanda, 13 districts had IPV prevalence of more than 50% for women and four districts reported high IPV in both women and men. Districts with high prevalence in the range of 54.2%-61% against women are scattered around Rwanda suggesting there may not be a global systematic spatial pattern. The negative global moran I value (Moran’s I = -0.1124, p = 0.76) for women IPV and (Moran’s I = -0.022, p = 0.456) for men indicates that IPV was not clustered together. However, the local indicators of spatial autocorrelation indicated some local clustering.

Tables S1 and S2 were used to inform the variables for inclusion in the adjusted generalized structured additive regression model. From the adjusted generalized structured additive regression model, the model with spatial random effects (DIC = 1788) was compared to the model without a spatial component (DIC = 1803) showing the relevance of adding the spatial component although the global Moran’s I was not significant. Table 3 shows the adjusted odds of IPV with corresponding credible intervals (CI) for the selected model.

**Table 3:** Bayesian Structured Additive Logistic Regression Model

	Women		Men	
Characteristics	aOR^*^	95% Credible Intervals	aOR	95% Credible Intervals
**Respondent age¥**	1.03	[1.01–1.05]	1.01	[0.99–1.03]
**Financial Decisions**				
Do not decide together	1			
Decide together	0.52	[0.40–0.67]	0.61	[0.41–0.93]
**Wealth Level**				
Poorest	1			
Poorer	0.81	[0.57–1.15]	0.86	[0.53–1.37]
Middle	0.89	[0.62–1.26]	0.77	[0.48–1.24]
Richer	0.49	[0.39–0.70]	0.61	[0.37-1.00]
Richest	0.48	[0.33–0.70]	0.54	[0.30–0.94]
**Partner controlling behaviour**				
No	1		1	
Yes	5.80	[4.55–7.39]	7.78	[5.35–11.52]
**Alcohol consumption**				
No	1		1	
Yes	3.11	[2.44–3.96]	2.31	[1.63–3.27]
**Random effects**				
Spatially structured effects [${f}_{str}\left({s}_{j}\right)$]	0.13	[0.00-0.65]	0.13	[0.00-0.99]
Spatially unstructured effects [${f}_{unstr}\left({s}_{j}\right)$]	0.07	[0.00-0.23]	0.21	[0.00-0.59]

^*^Mean posterior adjusted odds ratio estimates from the Bayesian structured additive model

^¥^Age of respondent was included as a non-linear covariate

Women making financial decisions together with their partner (aOR = 0.52; CI: 0.40–0.67), women in richer households (aOR = 0.49; CI: 0.39–0.70) and women in richest households (aOR = 0.48, CI: 0.33–0.70) are significantly at lower odds of experiencing IPV. In addition, women living with controlling partners (aOR = 5.80; CI: 4.55–7.39) and women with partners who consume alcohol (aOR = 3.11; CI: 2.44–3.96) are at significantly higher odds of experiencing IPV. In particular, women with controlling partners have about 6 times higher odds of experiencing IPV while those with partners that drink alcohol have about 3 times higher odds of experiencing IPV (Table 3).

Men who make financial decisions together with their partner (aOR = 0.61; CI: 0.41–0.93) and men in the richest households (aOR = 0.54; CI: 0.30–0.94)) are at significantly higher odds of experiencing IPV. In addition, men living with controlling partners (aOR = 7.78; CI: 5.35–11.52) and men with partners who consume alcohol (aOR = 2.31; CI: 1.63–3.27) are at significantly higher odds of experiencing IPV. In particular, men with controlling partners have about 8 times higher odds of experiencing IPV while those with partners that drink alcohol have about 2 times higher odds of experiencing IPV. Spatial correlation explains 64% of the residual spatial variation amongst women who experience IPV while in men experiencing IPV, 62% of the spatial variation is explained by district heterogeneity (Table 3).

**Fig. 2:**
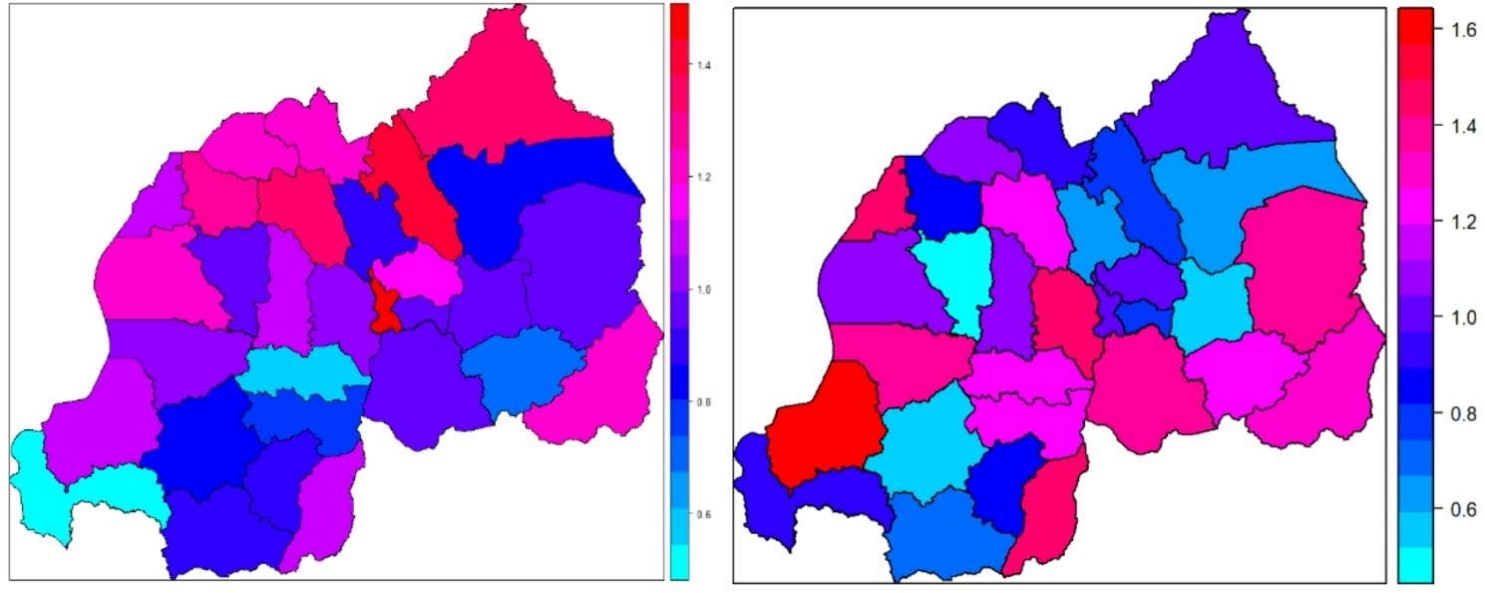
The predicted prevalence of IPV after adjusting for spatial as well as individual, partner and household level covariates among women (left) and men (right) in Rwanda

Figure 2 (left) shows elevated odds of IPV among women in Nyagatare, Gicumbi, Burera, Musanze, Nyabihu, Gakenke, Rutsiro, Nyarugenge, Gasabo and Kirehe districts which are mainly in the Northern province and Kigali after adjusting for selected individual, partner and household level covariates.

There are some pronounced IPV district-associated effects after adjusting for individual-level, partner as well as household-level covariates among women. The district-associated adjusted odds of IPV range from 0.56 to 1.40 with the structured spatial effects dominating the unstructured spatial heterogeneity effect as shown by the ratio ($\phi =\frac{{f}_{str}\left({s}_{j}\right)}{{f}_{str}\left({s}_{j}\right)+{f}_{unstr}\left({s}_{j}\right)}=\frac{0.132}{0.132+0.074}=64.1\%$).

Figure 2 (right) shows elevated odds of IPV among men in Gakenke, Rubavu, Karongi, Nyamasheke, Ruhango, Nyanza, Gisagara, Bugesera, Kamonyi, Ngoma, Kirehe and Kayonza districts which are mainly in the Eastern and Western provinces. The district-associated adjusted odds of IPV for men range from 0.52 to 1.60 with the spatial heterogeneity effects dominating the structured spatial effects as shown by the ratio ($\phi =\frac{{f}_{unstr}\left({s}_{j}\right)}{{f}_{str}\left({s}_{j}\right)+{f}_{unstr}\left({s}_{j}\right)}=\frac{0.211}{0.130+0.211}=62.0\%$). This indicates a general spatial variability in women perpetrated IPV against men in Rwanda with the Southern, Eastern and Western districts having elevated odds for men to experience IPV. Women experienced IPV show some level of structure than that experienced by men.

## Discussion

This study explores the spatial distribution, patterns, and associated factors of IPV against men and women using the 2019/2020 RDHS survey data, by fitting Bayesian structured additive logistic regression models. The study also uses districts administrative areas as spatial units, which is beneficial for policy decision-making especially for IPV.

We found a higher IPV prevalence for women (46%) than men (18%.) Our study is one of the very few studies that have reported men as victims of IPV [41]. Though the IPV prevalence in men is lower than females, it is relatively high and raises concern on the magnitude of IPV among men. The lower IPV prevalence in men can be explained by under-reporting of the information in the survey. Men may shy away from reporting as a ways for preserving their muscularity in the society. Moreover, women tend to have formal institutions which protect them; hence, they are normally better informed and feel more comfortable in reporting violence than men. There are many support services for women than men, therefore, it is important to involve the men in IPV campaigns and offer men appropriate support they may need so that they get the help they need like women. Though the recent SDG5.2.1 focuses on eliminating IPV among women and girls, there is silence on the men since this study has found that men are also affected with IPV regardless of the lower rates.

Our results are similar to those found in other studies [13], however our study highlights the specific districts in Rwanda where IPV prevalence is high for both women and men. Using a bivariate choropleth, IPV perpetrated against women was shown to be higher in the North-Western districts of Rwanda whereas for men it was shown to be more prevalent in the Southern districts. A few districts presented high IPV for both men and women. The study also found that reported IPV against women was almost twice that experienced by men. The lower prevalence in men can be explained by the African social norms. Men tend not to report being victims of IPV, especially if perpetrated by women as this may be construed as being weak [12–18, 42].

For IPV against women, physical violence was the most common (36.3%), followed by emotional violence (34.6%). Whereas, for men emotional violence (16.7%) was the most common. In line with our findings, a study in West Africa reported psychological or emotional violence being more commonly experienced among men [43]. This can be explained by the fact that men are more muscular and therefore do not usually suffer from physical violence perpetrated by their women intimate partners, whereas women tend to talk about their problems more than men and therefore are less likely to experience emotional violence.

Accounting for variability due to respondent age, how the couples made financial decisions, household wealth levels, partner controlling behavior and partner’s alcohol consumption, our study highlights districts that have elevated IPV prevalence. These districts are hotspots for IPV and the unobserved structural effects may need to be interrogated in order to mitigate against high IPV prevalence. We found higher odds of IPV against women mainly in the North-western districts and the spatial effects were dominated by spatially structured effects contributing 64%. On the other hand, higher odds of IPV were observed for men in the Southern districts of Rwanda and spatial effects were dominated by district heterogeneity accounting for 62%. There were no statistically significant district clusters for IPV in both men or women. Contrary to our findings, a study on IPV in Namibia [1], found a strong and significant clustering (structured component of IPV) mainly driven by poor economic conditions and cultural beliefs in the severely affected regions.

Rwanda is a more homogenous country with mostly Hutus and Tutsis constituting over 80% of the population, for which 75% reside in rural areas. Our results, adjusting for spatial effects, reveal that being in a higher socioeconomic status is protective to experiencing IPV for both women and men. This is consistent with results by [13, 43–46] who showed similar results using 2015 and 2020 DHS data. In addition, we also show that after adjusting for spatial effects, couples making financial decisions together are more protected against experiencing IPV. Several studies [44, 45] that have been conducted in Rwanda support this finding, including a clustered randomized trial study by [46] which highlighted that couples that discussed and completed tasks together had reduced experiences of IPV as a result of fostered higher relationship qualities. [46] also indicate that relationship quality is a key pathway for healthy behaviours for couples.

Our results show that women with higher education had lower odds of experiencing IPV and so were those married to partners with higher education. Making financial decisions together with a partner and living in the southern region were associated with lower odds of experiencing IPV in women. Women whose partners were of the same age, with controlling behaviour as well as alcohol consumers were at significantly higher odds of experiencing IPV. Similar findings were observed in men where higher education, those who were wealthier, and who made financial decisions together with their wives were associated with reduced risk of IPV. Our findings on the factors associated with IPV are in line with findings made by [13, 47–49].

The study did not fall short of some limitations, there was a lack of temporality between the factors and IPV, hence, we cannot ascertain causality of these factors on IPV. Not all potential factors were considered in the analysis, particularly in the Bayesian structured additive logistic regression model which is usually computationally intensive to attain convergency. Different variables were used for the male and female models which made it difficult to compare the effects between the two sub-populations. In addition, due to the nature of IPV being self-reported, men may have under-reported IPV perpetrated against them.

## Conclusion

This study found significant spatial variations in IPV against both men and women in Rwanda. Interventions that improve relationship quality within couples should be encouraged in the severely affected districts as well as across the country. The findings from our study show that making financial decisions together between partners helps in reducing intimate partner violence. Consumption of alcohol and unfavourable controlling behaviour by a partner may contribute to increase in IPV. There is a need to intensify campaigns that fight GBV and instill defense mechanism skills to individuals for future defense against GBV by their partners. Such campaigns and interventions should target both women and men if the SDG5 goal to eliminate IPV is to be achieved. Men cannot remain a blind spot in the fight of IPV.

Further research involving qualitative interviews with victims of IPV, both women and men, can provide a deeper understanding of drivers of IPV in Rwanda and similar African settings.

### Electronic supplementary material

Below is the link to the electronic supplementary material.

Supplementary Material 1

## Data Availability

The dataset generated and analysed during the current study are not publicly available since we received a data access letter from the DHS team https://dhsprogram.com/ specific to our project but are available from the DHS team upon request.
