# Supplementary material for: Bayesian spatial modelling of intimate partner violence and associated factors among adult women and men: evidence from 2019/2020 Rwanda Demographic and Health Survey
Source: BMC Public Health. 2023 Oct 20;23:2061. doi: 10.1186/s12889-023-16988-8 (PMC10589974; doi:10.1186/s12889-023-16988-8)
Supplement: Supplementary file 1 — Supplementary Material 1 [file 12889_2023_16988_MOESM1_ESM.docx]

Table S1. Factors associated with IPV women

|  | **Univariate analysis** | | | | **Multivariable/Adjusted analysis** | | |
| --- | --- | --- | --- | --- | --- | --- | --- |
| **Characteristics** | **%IPV** | **Crude OR**^a^ | **95%CI**^b^ | **p-value** | **Adjusted OR**^a^ | **95%CI**^b^ | **p-value** |
| **Socio-demographic** | | | | | | | |
| Age group, in years | | | | | | | |
| 15-24 | 45.3 | 1 |  |  | 1 |  |  |
| 25-34 | 44.8 | 0.88 | 0.61-1.26 | 0.478 | 0.91 | 0.57-1.44 | 0.686 |
| 35-49 | 46.9 | 0.95 | 0.66-1.37 | 0.796 | 1.03 | 0.64-1.68 | 0.890 |
| 50 above |  |  |  |  |  |  |  |
| Marital Status | | | | | | | |
| Not living together | 40.1 | 1 |  |  | 1 |  |  |
| Living with partner | 43.4 | 1.14 | 0.91-1.44 | 0.260 | 0.83 | 0.62-1.12 | 0.224 |
| Residence | | | | | | | |
| Rural | 42.4 | 1 |  |  | 1 |  |  |
| Urban | 46.6 | 0.84 | 0.65-1.09 | 0.197 | 1.26 | 0.86-1.84 | 0.237 |
| Highest education | | | | | | | |
| None | 54.1 | 1 |  |  | 1 |  |  |
| Primary | 48.1 | 0.79 | 0.58-1.06 | 0.116 | 0.95 | 0.64-1.39 | 0.784 |
| Secondary | 39.4 | 0.55 | 0.38-0.79 | 0.001 | 0.79 | 0.47-1.34 | 0.386 |
| Higher | 19.0 | 0.20 | 0.09-0.42 | <0.001 | 0.37 | 0.12-1.14 | 0.083 |
| Partner education |  |  |  |  |  |  |  |
| None | 49.9 | 1 |  |  | 1 |  |  |
| Primary | 43.5 | 0.77 | 0.55-1.08 | 0.141 | 0.92 | 0.61-1.38 | 0.671 |
| Secondary | 32.7 | 0.49 | 0.31-0.77 | 0.002 | 0.59 | 0.33-1.05 | 0.073 |
| Higher | 13.4 | 0.16 | 0.07-0.35 | <0.001 | 0.23 | 0.09-0.63 | 0.004 |
| Employed | | | | | | | |
| No | 42.2 | 1 |  |  | 1 |  |  |
| Yes | 64.6 | 1.19 | 0.89-1.61 | 0.243 | 1.34 | 0.91-1.97 | 0.134 |
| Wealth Level | | | | | | | |
| Poorest | 56.4 | 1 |  |  | 1 |  |  |
| Poorer | 49.1 | 0.75 | 0.55-1.01 | 0.058 | 0.96 | 0.63-1.47 | 0.863 |
| Middle | 46.7 | 0.68 | 0.49-0.92 | 0.013 | 0.92 | 0.61-1.41 | 0.709 |
| Richer | 39.8 | 0.51 | 037-0.71 | <0.001 | 0.73 | 0.48-1.11 | 0.139 |
| Richest | 37.7 | 0.47 | 0.33-0.66 | <0.001 | 1.01 | 0.59-1.71 | 0.963 |
| **Partner characteristics** | | | | | | | |
| Financial Decision | | | | | | | |
| No | 46.5 | 1 |  |  | 1 |  |  |
| Yes | 30.1 | 0.49 | 0.39-0.63 | <0.001* | 0.52 | 0.39-0.71 | <0.001 |
| Partner Control Behaviour |  |  |  |  |  |  |  |
| No | 21.5 | 1 |  |  | 1 |  |  |
| Yes | 68.1 | 7.79 | 6.14-9.89 | <0.001***** | 6.24 | 4.75-8.21 | <0.001 |
| **Pregnancy** |  |  |  |  |  |  |  |
| Currently Pregnant |  |  |  |  |  |  |  |
| No | 46.5 | 1 |  |  | 1 |  |  |
| Yes | 39.7 | 0.76 | 0.55-1.04 | 0.087 | 0.75 | 0.51-1.11 | 0.142 |
| **Age Difference** |  |  |  |  |  |  |  |
| Wife Older | 40.1 | 1 |  |  | 1 |  |  |
| Same Age | 53.4 | 1.71 | 1.07-2.75 | 0.025 | 2.14 | 1.21-3.78 | 0.009 |
| Husband Older | 46.2 | 1.28 | 0.95-1.73 | 0.106 | 1.04 | 0.73-1.46 | 0.842 |
| **Drinking** |  |  |  |  |  |  |  |
| Husband Drinking |  |  |  |  |  |  |  |
| No | 26.1 | 1 |  |  | 1 |  |  |
| Yes | 57.5 | 3.83 | 3.03-4.83 | <0.001 | 3.16 | 2.37-4.22 | <0.001 |
| Region |  |  |  |  |  |  |  |
| Kigali |  | 1 |  |  | 1 |  |  |
| South |  | 1.06 | 0.76-1.47 | 0.734 | 0.54 | 0.32-0.93 | 0.025 |
| West |  | 1.11 | 0.79-1.54 | 0.551 | 0.70 | 0.42-1.18 | 0.185 |
| North |  | 1.10 | 0.74-1.57 | 0.706 | 1.03 | 0.60-1.78 | 0.904 |
| East |  | 0.87 | 0.62-1.22 | 0.410 | 0.67 | 0.40-1.10 | 0.109 |

*****  p-value < 0.05,

^a^ Odds ratio,

^b^ 95% confidence interval

Table S2. Factors associated with IPV for men

| **Characteristics** | **Univariate analysis** | | | | **Multivariable/Adjusted analysis** | | |
| --- | --- | --- | --- | --- | --- | --- | --- |
|  | **%IPV** | **Crude OR**^a^ | **95%CI**^b^ | **P-value** | **Adjusted OR**^a^ | **95%Cl**^b^ | **p-value** |
| **Age group, in years** |  |  |  |  |  |  |  |
| 15 - 24 | 18.7 | 1 |  |  | 1 |  |  |
| 25 - 34 | 17.1 | 0.901 | 0.413 - 1.962 | 0.792 | 0.880 | 0.359 - 2.154 | 0.779 |
| 35 - 49 | 19.0 | 1.020 | 0.480 - 2.171 | 0.958 | 1.034 | 0.418 - 2.557 | 0.942 |
| 50 and above | 19.1 | 1.029 | 0.445 - 2.379 | 0.946 | 1.075 | 0.398 - 2.900 | 0.887 |
| **Marital Status** |  |  |  |  |  |  |  |
| Not living with partner | 18.6 | 1 |  |  | 1 |  |  |
| Living with partner | 18.1 | 0.970 | 0.693 - 1.360 | 0.861 | 1.018 | 0.676 - 1.533 | 0.933 |
| **Residence** |  |  |  |  |  |  |  |
| Rural | 17.7 | 1 |  |  | 1 |  |  |
| Urban | 18.6 | 0.943 | 0.609 - 1.461 | 0.792 | 1.186 | 0.641 - 2.195 | 0.586 |
| **Highest Education** |  |  |  |  |  |  |  |
| None | 23.6 | 1 |  |  | 1 |  |  |
| Primary | 18.8 | 0.751 | 0.474 - 1.188 | 0.220 | 0.755 | 0.442 - 1.289 | 0.302 |
| Secondary | 14.1 | 0.530 | 0.284 - 0.989 | 0.046* | 0.603 | 0.288 - 1.262 | 0.179 |
| Higher | 5.1 | 0.175 | 0.062 - 0.496 | 0.001* | 0.248 | 0.083 - 0.738 | 0.012* |
| **Employed** |  |  |  |  |  |  |  |
| No | 19.5 | 1 |  |  | 1 |  |  |
| Yes | 18.4 | 0.932 | 0.156 - 5.579 | 0.939 | 1 |  |  |
| **Wealth Level** |  |  |  |  |  |  |  |
| Poorest | 25.0 | 1 |  |  | 1 |  |  |
| Poorer | 18.7 | 0.694 | 0.421 - 1.142 | 0.150 | 0.634 | 0.351 - 1.144 | 0.13 |
| Middle | 20.3 | 0.768 | 0.482 - 1.225 | 0.267 | 0.679 | 0.397 - 1.160 | 0.156 |
| Richer | 12.4 | 0.425 | 0.248 - 0.727 | 0.002* | 0.365 | 0.188 - 0.705 | 0.003* |
| Richest | 15.5 | 0.552 | 0.320 - 0.952 | 0.033* | 0.746 | 0.362 - 1.537 | 0.426 |
| **Financial Decision** |  |  |  |  |  |  |  |
| No | 26.7 | 1 |  |  | 1 |  |  |
| Yes | 15.3 | 0.497 | 0.324 - 0.762 | 0.001* | 0.511 | 0.312 - 0.838 | 0.008* |
| **Control Behaviour** |  |  |  |  |  |  |  |
| No | 6.8 | 1 |  |  | 1 |  |  |
| Yes | 32.6 | 6.672 | 4.500 - 9.895 | 0.000* | 7.587 | 5.113 - 11.258 | 0.000* |
| **Wife current Pregnancy** |  |  |  |  |  |  |  |
| No or unsure | 17.9 | 1 |  |  | 1 |  |  |
| Yes | 10.9 | 0.559 | 0.281 - 1.110 | 0.096 | 0.622 | 0.297 - 1.303 | 0.208 |
| **Age Difference** |  |  |  |  |  |  |  |
| Women Older | 16.6 | 1 |  |  | 1 |  |  |
| Same Age | 14.6 | 0.857 | 0.477 - 1.541 | 0.605 | 0.978 | 0.495 - 1.932 | 0.949 |
| Man Older | 26.7 | 1.828759 | 1.243 - 2.691 | 0.002* | 1.412 | 0.847 - 2.354 | 0.185 |
| **Drinking** |  |  |  |  |  |  |  |
| Wife/partner drinking |  |  |  |  |  |  |  |
| No | 12.2 | 1 |  |  | 1 |  |  |
| Yes | 31.6 | 3.32 | 2.421 - 4.552 | 0.000* | 2.484 | 1.683 - 3.666 | 0.000* |
| **Region** |  |  |  |  |  |  |  |
| Kigali | 14.3 | 1 |  |  | 1 |  |  |
| South | 21.6 | 1.657 | 0.897 - 3.061 | 0.107 | 1.230 | 0.606 - 2.494 | 0.566 |
| West | 20.0 | 1.500 | 0.799 - 2.817 | 0.207 | 1.301 | 0.674 - 2.509 | 0.432 |
| North | 14.9 | 1.054 | 0.537 - 2.068 | 0.879 | 1.043 | 0.503 - 2.164 | 0.910 |
| East | 18.8 | 1.395 | 0.777 - 2.505 | 0.264 | 1.123 | 0.594 - 2.124 | 0.721 |

*****  p-value < 0.05,

^a^ Odds ratio,

^b^ 95% confidence interval
